# Supplementary material for: SyNDI: synchronous network data integration framework
Source: BMC Bioinformatics. 2018 Nov 6;19:403. doi: 10.1186/s12859-018-2426-5 (PMC6219086; doi:10.1186/s12859-018-2426-5)
Supplement: Supplementary file 5 — Files of the example presented in Additional file 4. This additional file is a zip package that contains all files used in Additional file 4. Detailed descriptions of each of these files are presented in a README file included in this zip package. (GZ 30411 kb) [file 12859_2018_2426_MOESM5_ESM.gz › SyNDITools/Meme2Fimo/meme_4.9.0_4.tar.gz!/meme_4.9.0/website/html/tomtom-intro.html]

Tomtom - Introduction


|  |  |
| --- | --- |
|  | MEME Suite The MEME Suite web application requires the use of JavaScript   Javascript doesn't seem to be available on your browser.  Introduction  Tomtom is a tool for comparing a DNA motif to a database of known motifs.  The Tomtom web application compares an input DNA motif to the elements of a database of known motifs (and their DNA reverse complements). A list of matching motifs is reported, ranked by q-value. The q-value is the minimal false discovery rate at which the observed similarity would be deemed significant. The output contains results for each query, in the order that the queries appear in the input file. With respect to each query, targets are ranked by q-value. For a given pair of motifs, the program considers all offsets, while requiring a minimum number of overlapping positions. For a given offset, each overlapping position is scored using one of seven column similarity functions defined below. In order to compute the scores, `Tomtom` needs to know the frequencies of the letters of the sequence alphabet in the database being searched (the "background" letter frequencies). By default, the background letter frequencies included in the MEME input files are used. The scores of columns that overlap for a given offset are summed. This summed score is then converted to a p-value. The reported p-value is the minimal p-value over all possible offsets. For each query motif, the corresponding p-values are then converted to q-values. Required Tomtom Inputs  Three inputs must be provided on the Tomtom web page:  1. A single DNA motif, with each column showing the counts    of bases A, C, G, and T, in that position of the motif.    The motif is pasted into the text box labeled "Paste your DNA here". 2. The DNA motif database to search.    This is selected from the drop-down list labeled "Select the DNA Motif    Database to Search". 3. The function used to compare motifs.    This is selected from the drop-down list labeled "Select the Motif Column    Comparison Function".   The contents of the required fields can be cleared by clicking on the "Clear Input" button. Submitting a Tomtom job  After filling in the required inputs on the Tomtom web page the job can be submitted for processing by clicking on the "Start Search" button. Alternatively, if you have no motif and would just like to see sample Tomtom output, you can just select the DNA motif databse, the motif column comparison function and then click on the "Search with Example Motif" button. Totom output  The result of submitting your job will be a page that displays the status of your job on the Tomtom server, and contains a link to your Tomtom results. The output of Tomtom is a web page displaying a table of motifs in the database similar to the motif you submitted (sample Tomtom output). Each entry in the table includes the database identifier for matching motif, its description, the p-value and q-value of the match, the overlap and offset between your motif and the matching motif, the strand of the matching motif, and a logogram of your motif and the matching motif. |
